# The Gut Microbiome in Heart Failure: Pathways to Inflammation and Therapeutic Targets

**DOI:** 10.3390/metabo16060431

**Published:** 2026-06-19

**Authors:** Uday Sankar Akash Vankayala, Ali Sohail, Bivin George, Madhu Singh, Omar Khayat, Malek Kreidieh, Alia Hasham, Luis Quiel

**Affiliations:** 1 Department of Internal Medicine, Staten Island University Hospital, Northwell Health, 475 Seaview Avenue, Staten Island, NY 10305, USA; asohail1@northwell.edu (A.S.); bgeorge22@northwell.edu (B.G.); msingh60@northwell.edu (M.S.); okhayat@northwell.edu (O.K.); lquiel@northwell.edu (L.Q.); 2 Division of Gastroenterology, Department of Internal Medicine, Staten Island University Hospital, Northwell Health, 475 Seaview Avenue, Staten Island, NY 10305, USA; mkreidieh@northwell.edu (M.K.); ahasham@northwell.edu (A.H.)

**Keywords:** gut microbiome, gut dysbiosis, heart failure, gut-heart axis, trimethylamine N-oxide (TMAO), short-chain fatty acids (SCFAs)

## Abstract

Heart failure (HF) continues to be a major global health burden, with persistent morbidity and mortality despite guideline-directed and device-based therapies. Evidence suggests the gut–heart axis is a critical and underrecognized contributor to HF progression. Alterations in cardiac output and systemic venous congestion in HF lead to intestinal hypoperfusion, mucosal edema, and loss of barrier integrity, increasing intestinal permeability, gut dysbiosis, and translocation of microbial products. This systemic translocation is associated with chronic low-grade inflammation that activates innate immune pathways that correlate with endothelial dysfunction, oxidative stress, fibroblast activation, and adverse cardiac remodeling. Gut-derived metabolites derived by microbial metabolism modulate cardiovascular health by altering the metabolic profiles. Dysbiosis results in loss of protective short-chain fatty acid (SCFA)-producing bacteria and enriches pro-inflammatory taxa such as trimethylamine N-oxide (TMAO)-producing bacteria. Elevated TMAO is associated with increased mortality and hospitalization in HF, whereas SCFAs enhance barrier integrity and immune tolerance. Secondary bile acids and uremic toxins such as indoxyl sulfate and p-cresyl sulfate further link dysbiosis to fibrosis and vascular stiffness. Circulating markers such as TMAO, lipopolysaccharide-binding protein (LBP), and soluble CD14 carry prognostic value beyond traditional cardiac biomarkers. This review highlights current experimental, translational, and clinical evidence describing gut dysbiosis and its molecular links to HF progression. Targeting the gut–heart axis represents a novel therapeutic approach in HF. Dietary modulation, probiotics/prebiotics, fecal microbiota transplantation, and inhibitors of microbial metabolic pathways show promise. Future research should emphasize microbiota-based interventions in HF management.

## 1. Introduction

Heart failure (HF) remains a global public health challenge, affecting more than 64 million individuals worldwide, and a leading cause of morbidity, mortality, and healthcare expenditures [1]. Despite major advances in guideline-directed medical therapy (GDMT) for heart failure and device-based interventions (implantable cardioverter-defibrillators, cardiac resynchronization therapy), the prognosis for many patients remains dismal with five-year survival rates around 50%, resembling that of cancer [2]. Aging populations, rising prevalence of hypertension, diabetes, and obesity have contributed to a growing HF epidemic in both high-income and low- to middle-income countries [3].

Patients with advanced or multimorbid phenotypes of HF, cardiorenal syndrome, and metabolic comorbidities show a suboptimal response to standard therapies. This prompts investigations to explore non-traditional pathogenic pathways of inflammation, such as gut microbiome-derived signaling, that may drive disease progression even under optimal management. Increasingly, the bidirectional communication between the gut and the heart, known as the “gut–heart axis,” is a recognized, yet underappreciated contributor to HF [4]. Compromised cardiac output and elevated venous pressures lead to intestinal hypoperfusion, congestion, and dysmotility. These changes disrupt the gut barrier integrity, leading to a “leaky gut” phenomenon and altering the gut microbiome. The gut-derived metabolites are implicated in systemic inflammation and cardiac remodeling, potentially creating a vicious cycle that exacerbates HF pathophysiology.

This review comprehensively examines bidirectional gut–heart interactions in HF, outlining key gut alterations during HF, pathways linking gut-derived metabolites to cardiac dysfunction, clinical utility of microbiome biomarkers, and current and emerging therapeutics targeting the gut–heart axis. This review generates a new outlook for understanding HF pathophysiology and outlines future directions in this field.

### 1.1. The Gut Microbiome: The Newly Recognized “Organ” and the Gut–Heart Axis

Over the past two decades, the gut microbiome has emerged as a functional organ system comprising trillions of bacteria, archaea, viruses, and fungi, collectively encoding more genes than the human genome itself. This complex ecosystem participates in host metabolism, immune education, and maintenance of intestinal integrity by producing metabolites such as short-chain fatty acids (SCFAs), secondary bile acids, and trimethylamine (TMA) [5]. Through these metabolites, the microbiome exerts systemic effects on nervous, renal, and cardiovascular systems.

**Figure 1:**
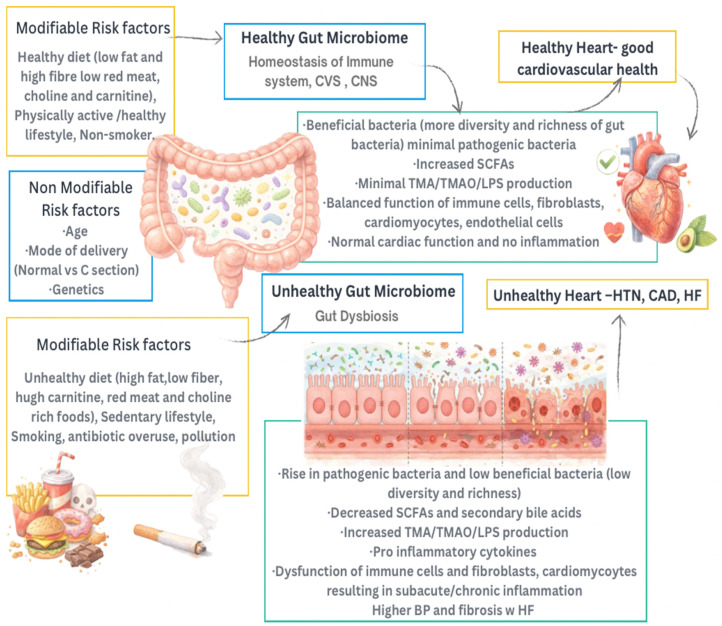
Schematic illustration showing how modifiable and non-modifiable risk factors influence gut microbiome composition and determine cardiovascular health.

In healthy individuals, symbiotic microbial communities maintain homeostasis and protect against colonization by pathogens. Disruption of microbial balance is termed “gut dysbiosis” and occurs due to several risk factors (illustrated in Figure 1); it has been linked to metabolic disorders, chronic kidney disease, and cardiovascular conditions, including atherosclerosis and hypertension. The HF disruption of this balance is termed gut dysbiosis, which can arise from dietary factors, antibiotics, comorbid diseases, and aging.

Evidence suggests that the microbiome is an active participant in cardiovascular regulation. This recognizes the gut microbiome as a potential therapeutic target and diagnostic biomarker within cardiovascular medicine.

The “gut–heart axis” refers to the bidirectional communication between the gastrointestinal tract and the cardiovascular system through immune, metabolic, neural, and endocrine pathways [6,7]. In HF cardiac dysfunction negatively impacts gut structure and function. Reduced cardiac output and elevated systemic venous pressures impair splanchnic perfusion and gut motility leading to cellular hypoxia, intestinal ischemia, mucosal edema congestion and anaerobic metabolism by gut bacteria. These hemodynamic derangements may potentially compromise the epithelial barrier integrity and disrupt the gut microbiome, resulting in increased intestinal permeability (“leaky gut”), dysbiosis, and translocation of bacteria and its microbial byproducts (e.g., lipopolysaccharide (LPS)) into the systemic circulation. This eventually triggers chronic inflammation, which may in turn aggravates cardiac remodeling and fibrosis, creating a vicious cycle of disease progression [8].

Microbial metabolites such as trimethylamine N-oxide (TMAO) promote atherogenesis and inflammation [9], whereas SCFAs including acetate, propionate, and butyrate exert anti-inflammatory and vasoregulatory effects, maintaining gut hemostasis [10]. This duality underscores the functional plasticity of the gut microbiome, and alteration can either amplify or mitigate cardiovascular injury depending on the overall microbial composition and host health status.

Clinical suggests elevated plasma TMAO levels are independently associated with increased mortality and rehospitalization in HF [11,12]. Conversely, interventions restoring microbial balance, such as dietary fiber enrichment, probiotics, or antimicrobial modulation, have shown evidence in improving endothelial function and cardiac outcomes in early clinical studies and settings. Together, these findings suggest the gut–heart axis as a novel pathophysiological link as well as a promising therapeutic frontier in the management of heart failure.

### 1.2. Objectives and Scope of the Review

Despite mounting evidence supporting the gut–heart interplay, a comprehensive synthesis of its mechanisms, clinical relevance, and therapeutic implications in HF remains limited. This review aims to comprehensively examine the bidirectional interactions between the gut and the failing heart. Specifically, it will outline the key alterations in the gut microbiome that occur during HF, determine the pathways through which gut-derived metabolites and immune mediators modulate cardiac function and influence clinical outcomes, And highlight the gut heart–axis and its emerging role as a biomarker of disease severity, prognosis, and therapeutic response. Finally, it will explore current and emerging therapeutic opportunities targeting the gut–heart axis and novel microbiome-directed pharmacologic strategies. By combining these dimensions, this review proposes new paradigms for HF pathophysiology and outlines future directions for translational research in this rapidly evolving field.

## 2. Methods

This narrative review synthesizes current evidence on the gut–heart axis in heart failure through a comprehensive literature search. We searched PubMed/MEDLINE, Embase, Web of Science, and Scopus databases from inception through December 2025.

Search terms combined Medical Subject Headings (MeSH) and free-text keywords across three domains:

1. Heart failure (“heart failure” OR “cardiac failure” OR “HFrEF” OR “heart failure with reduced ejection fraction” OR “HFpEF” OR “heart failure with preserved ejection fraction” OR “cardiac dysfunction” OR “congestive heart failure” OR “ventricular dysfunction”);
2. Gut microbiome (“gut microbiota” OR “gut microbiome” OR “intestinal microbiota” OR “intestinal microbiome” OR “dysbiosis” OR “gut flora” OR “gastrointestinal microbiome” OR “fecal microbiota”);
3. Mechanisms/metabolites (“TMAO” OR “trimethylamine N-oxide” OR “trimethylamine” OR “short-chain fatty acids” OR “SCFA” OR “butyrate” OR “propionate” OR “acetate” OR “bacterial translocation” OR “intestinal permeability” OR “leaky gut” OR “lipopolysaccharide” OR “LPS” OR “endotoxemia” OR “gut-heart axis” OR “microbiome-derived metabolites” OR “bile acids” OR “uremic toxins”).

Boolean operators (AND, OR) connected terms across domains. Reference lists of key articles were manually screened for additional relevant studies.

### Study Selection and Inclusion Criteria

As a narrative review, study selection prioritized relevance to review objectives rather than strict systematic criteria. We included peer-reviewed original research (human and animal studies), clinical trials, observational studies, and high-quality systematic reviews examining gut microbiome alterations, gut-derived metabolites (TMAO, SCFAs, bile acids, uremic toxins), gut–heart crosstalk, and cardiovascular outcomes in heart failure. Publications were limited to the English language. Conference abstracts, editorials, and studies lacking methodological clarity were excluded.

**Figure 2:**
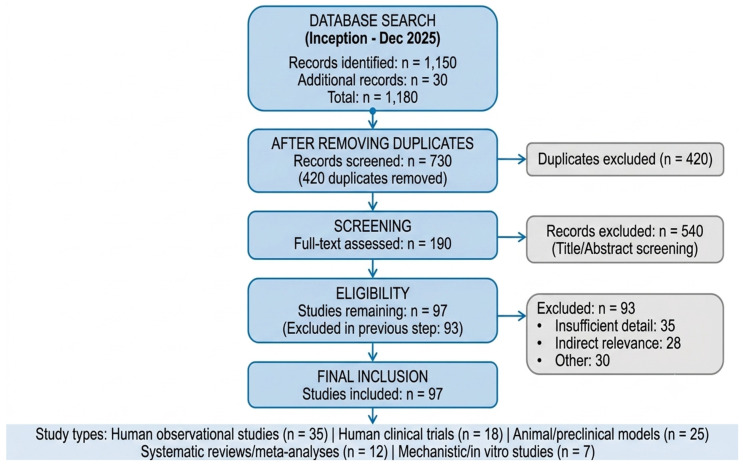
Literature search and study selection process.

The initial search identified approximately 1150 articles. After duplicate removal (*n* = 420) and title/abstract screening of 730 unique records. Two independent reviewers (USAV, AS) screened titles and abstracts for relevance based on inclusion criteria. Full texts of potentially relevant articles were retrieved and assessed for eligibility. Disagreements were resolved through discussion with senior authors (MK, AH). Ninety-seven studies were finally selected for narrative synthesis based on scientific rigor, relevance, and contribution to understanding the gut–heart axis. While narrative rather than systematic, coverage was ensured through multi-database searching, reference tracking, and prioritization of recent publications alongside seminal earlier studies (Figure 2).

Data were synthesized qualitatively across five thematic domains: hemodynamic gut alterations in HF, mechanistic pathways of gut–cardiac crosstalk, gut-derived metabolites and biomarkers, clinical prognostic implications, and therapeutic interventions.

Flow diagram illustrating the systematic identification, screening, and selection of studies for this narrative review. Database searches of PubMed/MEDLINE, Embase, Web of Science, and Scopus were conducted from inception through December 2025. After duplicate removal and systematic evaluation, 97 studies were included in the final synthesis.

## 3. Heart Failure-Induced Changes in the Gut Environment

### 3.1. Gut Hypoperfusion and Ischemia

The gastrointestinal tract receives up to 25% of resting cardiac output and is vulnerable during low-flow states [13]. Reduced cardiac output in HF diminishes splanchnic perfusion, leading to hypoxia and ischemia of the intestinal mucosa. Sustained hypoperfusion activates hypoxia-inducible pathways, generates oxidative stress, and promotes epithelial apoptosis, collectively impairing intestinal barrier integrity. This disrupts the epithelial integrity and mucosal architecture, causing loss of tight junction proteins (occludins and claudins), widening of paracellular spaces, and villous blunting rendering the gut more permeable and vulnerable to luminal insults [8]. Patients with chronic heart failure demonstrate significantly elevated lactulose-to-mannitol (L/M) ratios, reflecting increased intestinal permeability and impaired absorptive capacity. The L/M ratio has emerged as a validated, noninvasive marker of gut barrier dysfunction that correlates with disease severity and reduced cardiac index [14].

Experimental animal models [15,16,17,18] confirm these observations. Low-flow states induced by hemorrhagic shock, aortic banding, or cardiogenic shock consistently result in epithelial tight junction disruption, increased endotoxin translocation, and systemic inflammatory activation. These models demonstrate that intestinal hypoperfusion alone is sufficient to permit bacterial translocation to mesenteric lymph nodes, liver, and systemic circulation, providing a basis for inflammation and immune activation observed in HF.

### 3.2. Intestinal Congestion and Edema

Systemic venous congestion, is the hallmark of decompensated HF. Elevated central venous and portal pressures are transmitted directly to the splanchnic circulation and cause intestinal wall edema and increased interstitial pressures within the mucosa and submucosa. Compression of the lymphatic vessels impairs lymphatic drainage, augmenting congestion. This mechanical stress distorts villi and crypt architecture and disrupts epithelial tight junctions, compromising the barrier integrity and increasing paracellular permeability [18].

At the molecular level, elevated venous pressure triggers specific signaling cascades that dismantle tight junction complexes. Mechanical stretch activates the RhoA GTPase/Rho-associated protein kinase (ROCK) pathway, which phosphorylates myosin light chain (MLC) and promotes cytoskeletal contraction. This disrupts the tight junction assembly and increases paracellular permeability [19,20]. Simultaneously, hypoxia upregulates matrix metalloproteinases (MMPs), particularly MMP-2 and MMP-9, that causes the proteolysis of occludins and claudins proteins [21]. Oxidative stress from intestinal ischemia–reperfusion injury activates protein kinase C (PKC) pathways that phosphorylate tight junction proteins, triggering their internalization and elimination from the cell membrane [22]. Inflammatory mediators such as tumor necrosis factor-α (TNF-α) and interferon-γ (IFN-γ) signal through nuclear factor-κB (NF-κB) to suppress tight junction gene transcription while upregulating myosin light chain kinase (MLCK), further destabilizing the barrier [23,24]. Collectively, these pathways represent active, signal-mediated disassembly rather than passive mechanical failure.

Experimental models confirm this theorum of dual hemodynamic insults of venous congestion and arterial hypoperfusion affecting occludin, claudin-4, and ZO-1 localization [25,26,27]. Venous congestion also slows intestinal motility through bowel wall edema and autonomic dysfunction, creating a favorable environment for small intestinal bacterial overgrowth (SIBO). Bacterial overgrowth increases luminal endotoxin burden and promotes translocation of lipopolysaccharide (LPS) and other pathogen-associated molecular patterns (PAMPs) across the compromised epithelium, contributing to systemic inflammation and immune activation in HF [27,28]. Sandek et al. [27] demonstrated significantly increased bowel wall thickness across multiple intestinal segments in patients with chronic HF compared with controls. Mean wall thickness was increased in all segments of the bowel [terminal ileum (1.48 ± 0.16 mm vs. 1.04 ± 0.08 mm), ascending colon (2.32 ± 0.18 mm vs. 1.31 ± 0.14 mm), transverse colon (2.19 ± 0.20 mm vs. 1.27 ± 0.08 mm), descending colon (2.59 ± 0.18 mm vs. 1.43 ± 0.13 mm), and sigmoid colon (2.97 ± 0.27 mm vs. 1.64 ± 0.14 mm)] (all *p* < 0.01), reinforcing edema and congestion.

### 3.3. The Result: Gut Dysbiosis in Heart Failure

Multiple human and animal studies [29,30,31] reveal a characteristic alteration in gut microbial communities in HF, often referred to as an “HF microbiome signature.” The resultant cellular hypoxia, vascular congestion, and over expression of sodium/hydrogen exchanger lead to increased sodium transport, lowering the luminal pH and shifting the composition of the gut microbiome. Metagenomic analyses reveal a reduction in microbial alpha diversity and overall richness, a depletion of beneficial short-chain fatty acid-producing taxa such as *Faecalibacterium prausnitzii*, *Roseburia*, *Eubacterium*, and bacteriodes/bifidobacteria, And the enrichment of enteropathogenic organisms—members of the *Enterobacteriaceae* family and *Escherichia*/*Shigella* [30]. At the phylum level, several cohorts have reported an increased Firmicutes-to-Bacteroidetes ratio that suggests a loss of fermentative and short-chain fatty acid-producing pathways and favors lipopolysaccharide (LPS) biosynthesis, creating a pro-inflammatory profile. The severity of this dysbiosis has been shown to correlate with clinical phenotypes of the New York Heart Association (NYHA) functional class and higher circulating natriuretic peptide levels, thus supporting the casual link between gut microbial perturbations and HF progression.

## 4. Key Mechanisms of the Gut–Heart Axis

### 4.1. Increased Intestinal Permeability (“Leaky Gut”) and Systemic Inflammation

Disruption of intestinal epithelium and tight junctions permits translocation of luminal microbial products, with luminal antigens (e.g., LPS, peptidoglycans, flagellin and bacterial DNA) gaining access to the lamina propria and enabling systemic circulation. This is the “leaky gut” hypothesis that results in chronic endotoxemia. Translocated LPS binds circulating LPS-binding protein (LBP) and CD14, activating Toll-like receptors (e.g., TLR4) on innate immune cells, vascular endothelium, and cardiomyocytes.

Activation of the TLR4–MyD88–NF-κB pathway [32,33] induces sustained production of pro-inflammatory cytokines, including interleukin-6 (IL-6), TNF-α, and interleukin-1β (IL-1β) [34]. These cytokines exert pleiotropic effects relevant to HF pathophysiology-promoting endothelial dysfunction via nitric oxide imbalance, upregulating adhesion molecules (ICAM-1, VCAM-1), increasing oxidative stress through NADPH oxidase activation, and stimulating fibroblast proliferation and extracellular matrix deposition within the myocardium. Over time, these processes contribute to adverse ventricular remodeling, myocardial fibrosis, and progressive vascular stiffness [27]. Subsequent experimental models have reinforced this paradigm. Experimental models in mice confirm that circulating LPS and LPS-binding protein levels promote TLR4 signaling that directly contributes to inflammation and worse outcomes. On the contrary, TLR4 knockout mice are protected from pressure overload-induced cardiac remodeling [35,36,37]. The higher circulating LPS and LBP in HF patients, compared with controls, strongly correlate with inflammatory markers (CRP, IL-6), disease severity (NYHA class), and adverse clinical outcomes, including hospitalization and mortality [37,38]. In HF patients, endotoxemia is not transient but chronic, contributing to sustained immune activation. This may represent the gut-derived immune mediators as a non-traditional driver of HF pathophysiology.

### 4.2. Microbial Metabolites as Key Mediators

#### 4.2.1. Trimethylamine N-Oxide: The Pro-Atherogenic Culprit

TMAO is one of the most intensively studied gut-derived metabolites in cardiovascular disease. TMAO is generated from dietary precursors (e.g., choline, phosphatidylcholine, L-carnitine) that are metabolized by gut bacteria to trimethylamine (TMA). In the liver, flavin monooxygenases (predominantly FMO3) oxidize TMA to TMAO [9]. TMAO exerts systemic effects on cholesterol and bile acid metabolism by impairing reverse cholesterol transport, reducing bile acid synthesis, and altering hepatic sterol metabolism. It enhances platelet function, resulting in heightened thrombosis risk and inflammation. At the vascular level, TMAO promotes endothelial dysfunction through reduced nitric oxide bioavailability, increased oxidative stress, and upregulation of adhesion molecules, fostering a pro-inflammatory and pro-atherothrombotic milieu [39]. Studies demonstrated that suppression of gut microbiota using broad-spectrum antibiotics nearly abolishes plasma TMAO production following a phosphatidylcholine challenge, confirming the obligatory role of intestinal microbes in this pathway [40].

In HF settings, elevated TMAO levels are associated with higher mortality, more frequent hospitalizations, and worse cardiac remodeling [41]. Tang et al. [42] demonstrated that individuals who experienced major adverse cardiovascular events (MACE) had significantly higher baseline TMAO levels compared with those without events (median 5.0 μM vs. 3.5 μM; *p* < 0.001). Elevated TMAO levels were independently associated with a more than 3× increased risk of all-cause mortality and a 2× increased risk of nonfatal myocardial infarction or stroke, even after adjustment for traditional cardiovascular risk factors and renal function. This correlates with adverse ventricular remodeling, increased myocardial fibrosis, higher New York Heart Association (NYHA) functional class, and greater rates of hospitalization and mortality. Experimental evidence suggests TMAO directly promotes myocardial fibrosis through activation of pro-fibrotic pathways, particularly transforming growth factor-β (TGF-β)/Smad signaling, leading to enhanced cardiac fibroblast proliferation and collagen deposition. TMAO impairs mitochondrial function and amplifies inflammation via activation of nuclear factor-κB (NF-κB) pathways and upregulation of inflammatory proteins [monocyte chemoattractant protein-1 (MCP-1), macrophage inflammatory protein-2 (MIP-2),TNF-α, adhesion molecules (ICAM-1, VCAM-1), E-selectin, cyclooxygenase-2, and CD68], leading to cardiac fibroblast proliferation and collagen deposition [43]. This role of TMAO is supported by animal models where pharmacologic inhibition of microbial trimethylamine (TMA) formation using TMA-lyase inhibitors, such as 3,3-dimethyl-1-butanol, significantly reduces circulating TMAO levels and attenuates myocardial fibrosis and inflammation in heart failure models [44].

Collectively, these data suggest TMAO as a critical mechanistic link between gut dysbiosis and adverse cardiac remodeling that can be further validated through interventional trails. Nonetheless, TMAO represents a promising therapeutic target for improving cardiovascular outcomes.

#### 4.2.2. Short-Chain Fatty Acids: The Protective Metabolites

Short-chain fatty acids (SCFAs), primarily acetate, propionate and butyrate, are microbial metabolites that arise from bacterial fermentation of dietary fiber [45]. They contribute to gut and systemic homeostasis by enhancing epithelial barrier integrity through upregulation of tight junction proteins (claudins and occludins) and mucus production by goblet cells [46]. Butyrate, in particular, serves as the primary energy source for the colonic epithelium, promoting epithelial repair and exerting anti-inflammatory effect [47,48]. SCFAs exert potent immunomodulatory effects by promoting the differentiation of regulatory T cells (Tregs) via histone deacetylase (HDAC) inhibition and G protein-coupled receptor signaling (GPR41, GPR43). SCFAs suppress pro-inflammatory cytokine production such as TNF-α, IL-6, and IL-1β. This shift toward immune tolerance mitigates chronic low-grade inflammation, supporting endothelial function by enhancing nitric oxide bioavailability and reducing oxidative stress, thereby protecting against microvascular dysfunction the central driver for heart failure [47,49]. Through activation of GPR41 and GPR43 expressed on vascular endothelium, renal tissue, and sympathetic ganglia, SCFAs contribute to blood pressure regulation, dampen sympathetic nervous system activity, and modulate renin–angiotensin–aldosterone signaling, influencing systemic hemodynamic and neurohormonal regulation [50,51]. This highlights their cardioprotective role by limiting adverse cardiac outcomes.

Multiple studies showed a consistent reduction in SCFA-producing bacteria in HF patients, particularly *Faecalibacterium prausnitzii*, *Roseburia*, and *Eubacterium* species, and decreased SCFA levels in HF patients [52,53,54]. This reduced circulating and fecal SCFAs correlates with impaired endothelial function, higher New York Heart Association (NYHA) functional class, and worse clinical outcomes. Loss of SCFA-mediated barrier protection drives inflammatory and neurohormonal imbalances in HF progression [55].

#### 4.2.3. Other Metabolites

Beyond well-characterized gut-derived metabolites such as SCFA and TMAO, studies suggest that several non-canonical microbial mediators may also contribute to gut–heart crosstalk. Secondary bile acids produced through microbial deconjugation and metabolism of primary bile acids represent an important class of signaling molecules. Bile acids modulate signaling via the farnesoid X receptor (FXR) and the G protein-coupled bile acid receptor TGR5, which in turn regulate lipid and glucose metabolism, mitochondrial energetics, inflammatory signaling, and endothelial function [41]. Models demonstrated dysregulated bile acid profiles and FXR/TGR5 signaling modulates myocardial hypertrophy, fibrosis, and systemic metabolic homeostasis, contributing to HF progression [56]. The bile acid pool composition may serve as a fingerprint of microbiome function and a modifiable factor in cardiovascular patients.

Tryptophan undergoes microbial metabolism and generates derivatives that play a divergent role in HF. Clostridium and Firmicutes metabolize tryptophan and produce Indole-3-propionic acid (IPA), which has a potent cardioprotective effect and activates aryl hydrocarbon receptor (AhR), promoting intestinal barrier integrity and suppressing inflammation through powerful antioxidant properties [57]. Studies demonstrate that higher serum IPA predicts lower risk of type 2 diabetes and major adverse cardiovascular events (MACE), though dedicated HF studies are absent [58]. On the other hand, uremic toxins such as indoxyl sulfate and p-cresyl sulfate are another critical group of microbiota-derived metabolites that play a role in heart failure patients, especially those with concomitant renal dysfunction. These toxins, produced from bacterial metabolism of dietary tryptophan and tyrosine, accumulate due to reduced renal clearance and enhanced intestinal permeability. These toxins promote oxidative stress, endothelial dysfunction, vascular stiffness, and myocardial fibrosis through activation of pro-inflammatory and pro-fibrotic pathways, including NF-κB, AhR, etc. [44].

Imidazole propionate (ImP) is another metabolite linking gut dysbiosis to cardiometabolic dysfunction. It is produced by bacterial histidine fermentation in dysbiotic states and impairs insulin signaling through p38γ MAPK and mTORC1 activation [59]. Elevated plasma ImP has been implicated in insulin resistance, and emerging evidence suggests correlation with diastolic dysfunction markers in HFpEF population [60,61].

Polyamines, phenyl derivatives, and aromatic amino acid-derived compounds, also recognized as modulars of cardiovascular physiology, may further influence vascular tone, inflammatory signaling, and cardiometabolic regulation [45]. Polyamines such as putrescine, spermidine, and spermine influence mitochondrial function and cellular senescence, with evidence suggesting cardioprotective effects under physiologic conditions but maladaptive consequences when dysregulated [62,63]. However, their precise roles in HF remain incompletely defined.

Several other microbial metabolites warrant attention. Branched-chain amino acid (BCAA) metabolites from incomplete catabolism promote insulin resistance and accelerate HF [64]. Choline-derived metabolites include betaine with cardioprotective effects and γ-butyrobetaine predicting cardiovascular events comparable to TMAO [65]. Phenylacetylglutamine independently predicted MACE through enhanced platelet reactivity in large cohorts [66]. Hydrogen sulfide from sulfate-reducing bacteria exhibits hormetic effects, while dietary nitrate undergoes bacterial reduction to nitric oxide, with trials demonstrating improved exercise capacity in HF through beetroot juice supplementation [67,68]. Equol, a soy isoflavone metabolite produced in only 30–50% of individuals, has been found to have superior cardiovascular benefits [69].

Overall, these non-canonical mediators expand the conceptual framework of the gut–heart axis.

**Figure 3:**
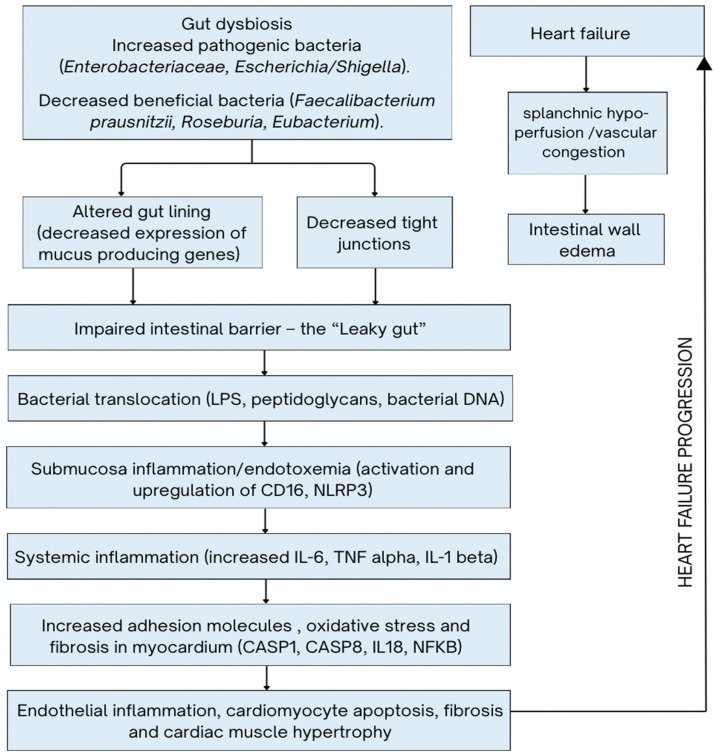
A schematic illustration showing the bidirectional interaction between heart failure and gut dysbiosis. The cycle of gut dysbiosis, intestinal barrier breakdown, triggering of systemic inflammation, and progression of heart failure.

A summary of this interaction between gut microbiome and its derivatives in HF pathophysiology is illustrated in Figure 3.

Table 1 Summarizes the major clinical studies investigating gut-derived metabolites and microbial biomarkers in heart failure.

## 5. Clinical Implications of the Gut–Heart Axis

Evidence indicates that gut-derived signals not only participate in HF pathogenesis but also carry prognostic value beyond conventional cardiovascular biomarkers of N-terminal pro-B type natriuretic peptide (NT-proBNP), renal function (eGFR), etc. TMAO has emerged as one of the most robustly studied microbial metabolites. Several chronic and acute HF cohorts have demonstrated that plasma TMAO levels independently predict adverse clinical outcomes, including all-cause mortality and HF-related hospitalization [36]. Circulating markers such as LPS-binding protein [70,71] and soluble CD14, reflecting chronic low-grade bacterial translocation, have been shown to correlate with inflammatory burden, HF severity, and worse prognosis [72]. Recent studies using 16S rRNA sequencing and metagenomic profiling have identified characteristic shifts in microbial composition called the “HF microbiome signature”, characterized by a loss of anti-inflammatory, butyrate-producing bacteria like *Faecalibacterium prausnitzii*. This has been associated with heightened systemic inflammation and worse clinical outcomes in HF cohorts [73]. These gut-derived biomarkers provide a nuanced basis for HF prognostication.

While both heart failure with reduced ejection fraction (HFrEF) and heart failure with preserved ejection fraction (HFpEF) phenotypes exhibit gut dysbiosis and elevated TMAO, HFpEF profiles are often confounded by metabolic comorbidities like obesity and diabetes [74,75]. Further research is needed to determine if the gut–heart axis differs across phenotypes, which would allow for more precise, phenotype-specific biomarkers and therapies.

## 6. Therapeutic Strategies Targeting the Gut Microbiome in Heart Failure

### 6.1. Dietary Interventions

Dietary modification is a foundational and low-risk strategy to influence gut microbe and metabolite production. Increased intake of dietary fiber, components of a Mediterranean diet and plant-rich diets, favors SCFA-producing taxa, enhances epithelial barrier integrity, and reduces substrate availability for TMAO production [76,77]. Conversely, high consumption of red meat, choline-rich foods, and excess saturated fat increases the availability of microbial TMA precursors and, in turn, TMAO production [78]. Dietary restriction of these components has been proposed as a strategy to attenuate gut-derived pro-inflammatory signaling.

Certain bioactive dietary compounds may exert targeted effects. Resveratrol (RSV), a naturally occurring polyphenolic phytoalexin found in grapes, berries, and red wine, has been shown to reduce circulating TMAO levels. Chen et al. [79] demonstrated that RSV supplementation suppresses TMA/TMAO formation by inhibiting the L-carnitine-to-TMA pathway while simultaneously enriching beneficial genera such as *Lactobacillus* and *Bifidobacterium*. This highlights dietary polyphenols as promising adjunctive modulators of the gut–heart axis.

### 6.2. Probiotics and Prebiotics

Probiotics (*Lactobacillus*, *Bifidobacterium*, and *Saccharomyces* species) and prebiotics (inulin and fructooligosaccharides) aim to restore gut microbial balance by introducing beneficial bacterial strains or promoting their growth. Studies in HF patients have shown probiotic formulations to shift the SCFA profiles [80]. Preclinical models showed cardioprotective signals with probiotic supplementation. Supplementation of antibiotic-treated mice with a probiotic containing *Lactobacillus plantarum* and *Bifidobacterium lactis* prior to myocardial infarction shifted the host SCFA balance to propionic acid, resulting in cardioprotective effects [81,82]. Reduced left ventricular hypertrophy (LVH), improvements in ejection fraction (EF), and minimal alteration of the overall microbiome were observed. Human studies data are mixed due to small samples, heterogeneity, and short follow-ups. Larger RCTs with standardized endpoints are needed.

### 6.3. Fecal Microbiota Transplantation

Fecal microbiota transplantation (FMT) represents a radical approach to reset gut microbiome composition. Animal studies in mice suggest that FMT from healthy donors can ameliorate cardiac remodeling and reduce inflammation, supporting the plausibility of microbiome-based therapies [83]. Recent human trials in metabolic syndrome provide cautious optimism. Kootte et al. [84] showed that lean-donor FMT improved insulin sensitivity in 38 recipients with obesity at 6 weeks, though benefits were transient with return to baseline by 18 weeks, while Vrieze et al. [85] demonstrated similar improvements in 18 men, with responders characterized by higher baseline microbial diversity. Safety profiles show predominantly mild gastrointestinal side effects, though a 2019 report documented fatal drug-resistant Escherichia coli transmission via FMT, prompting enhanced screening protocols [86].

However, application to HF remains theoretical given safety concerns, donor selection, long-term durability of microbial engraftment, and infectious risks specially in a population with advanced age and comorbidities. Though no published randomized trials exist in HF, a couple of pilot studies are underway and are expected to achieve phase 1 completion by 2027.

FMT is a transformative proof of concept warranting future investigations and studies.

### 6.4. Novel Pharmacological Approaches

Targeting gut microbiome and its metabolic pathways represent a novel therapeutic avenue in HF management. Among the most promising strategies are inhibitors of microbial TMA production. Bacterial TMA lyases, encoded by genes such as *cutC/D*, catalyze the conversion of dietary choline and L-carnitine into TMA, the precursor of TMAO [44]. Preclinical models of these inhibitors have shown to markedly reduce circulating TMA and TMAO levels without broadly suppressing the gut microbiota. Structural analogs such as 3,3-dimethyl-1-butanol (DMB), a naturally occurring compound found in certain dietary oils and vinegars, have demonstrated potent inhibition of microbial TMA formation. In animal models, DMB supplementation significantly reduced TMAO levels, attenuated vascular inflammation, limited myocardial fibrosis, and improved cardiac remodeling [87]. Prebiotic-like small molecules selectively promote short-chain fatty acid (SCFA)-producing taxa or suppress pro-inflammatory bacterial populations, thus restoring the gut microbiome balance [88,89]. Barrier-supportive agents enhance tight-junction integrity, mucosal healing, and antioxidant defenses to mitigate congestion and ischemia-induced permeability in HF.

Despite the compelling data, translation of these novel strategies into human trails is limited. The findings provide a strong proof of concept that selectively targeting microbial metabolic outputs can favorably modulate host cardiovascular outcomes, representing a promising frontier in HF therapeutics [90].

## 7. Gaps in Knowledge and Limitations

Despite strong plausibility, several important gaps remain. First, causality vs. association remains the fundamental challenge. Most human evidence linking gut dysbiosis and HF outcomes is observational, limiting causal inference and leaving the question of whether microbiome alterations are primary drivers of HF progression or secondary epiphenomena, reflecting advanced disease severity, unresolved [91]. While these cross-sectional and cohort studies establish correlations between gut dysbiosis, elevated TMAO, endotoxemia, and adverse HF outcomes, it does not establish directionality or exclude confounding. Several complementary approaches could strengthen causal inference in future research. Mendelian randomization (MR) could assess causality while minimizing confounding and reverse causation. For example, genetic variants in FMO3 could determine whether genetically elevated TMAO causally increases HF risk. MR analyses have suggested potential causal links between gut microbiome features and coronary artery disease and metabolic disorders [92], though applications in HF remain unexplored. Longitudinal cohort studies with serial microbiome profiling, ideally beginning before HF onset, could determine whether dysbiosis precedes or follows disease development while accounting for time-varying confounders including diet, antibiotics, and diuretics. Existing cohorts predominantly capture prevalent rather than incident HF, limiting causal inference. Interventional studies such as randomized controlled trials with microbiome-directed interventions such as dietary fiber, probiotics, TMAO-lowering agents, or fecal microbiota transplantation would establish therapeutic causality and impact on cardiac outcomes. Current trials are limited to small cohorts, shorter duration, and focus on surrogate rather than clinical endpoints. Experimental animal models of germ-free mice colonized with defined consortia, metabolite supplementation, and genetic receptor deletion (TLR4, GPR41/43) provide a proof of concept, but require human validation given species differences in microbiome composition and cardiac physiology [93]. Until such multilevel evidence accumulates, we must carefully distinguish observational association from biologically plausible mechanism and established causation.

Second, heterogeneity exists across cohorts, with microbiome signatures varying by geography, diet, ethnicity, comorbidities, and sequencing methodologies, complicating reproducibility and biomarker standardization [94]. Methodological heterogeneity across microbiome studies complicates reproducibility. Studies may employ different sequencing approaches (16S rRNA versus shotgun metagenomics), bioinformatics pipelines (QIIME, mothur, DADA2), taxonomic databases (Greengenes, SILVA, RDP), and pre-analytical protocols (sample storage, DNA extraction methods, sequencing depth), yielding divergent results from identical samples. Standardization of microbiome research methods is critical for advancing research in cardiovascular disease.

Third, the temporal dynamics of microbiome changes in HF, particularly their responsiveness to disease progression or GDMT, remain poorly characterized, as longitudinal studies are limited. Interventional uncertainty also persists. While FMT or microbial enzyme inhibition show promise in preclinical models, their long-term safety, durability, potential off-target effects, and microbiome resilience needs careful study in humans [83]. Differences across phenotypes of heart failure with reduced ejection fraction (HFrEF), preserved ejection fraction (HFpEF) and right-sided HF are understudied, which limits our understanding of the response of therapeutic strategies across phenotypes [95,96].

Notably our review is only restricted to English-language literature and may therefore omit relevant findings from non-English publications. As a narrative review, selection bias and heterogeneity across studies is possible.

## 8. Conclusions and Future Directions

The gut microbiome and intestinal barrier integrity have emerged as key players in the pathophysiology of heart failure, yet they are underrecognized. Intestinal hypoperfusion and congestion, epithelial barrier disruption, microbial translocation, and metabolite signaling via TMAO and SCFAs result in systemic inflammation and adverse cardiac outcomes. This reinforces how a gut–heart feedback loop may change the course of the disease. Targeting this axis may offer novel diagnostic and therapeutic strategies adjunctive to conventional HF care.

Future progress in this field will require a shift from observations to interventional science. Large, prospective cohorts with longitudinal sampling are required to define causal trajectories and temporal relationships of microbiome changes in heart failure and its phenotypes. Randomized controlled trials (RCTs) test dietary modulation, prebiotics, probiotics, and microbial enzyme-based interventions in HF populations. FMT and next-generation microbiota-targeted therapeutics are required to test feasibility and clinical efficacy in HF. Future human studies, leveraging endotoxemia models and isotope-based metabolic flux assays, are essential for identifying critical host microbe pathways amenable to intervention. Ultimately, developed, personalized approaches to integrating microbiome data with host genomics to tailor therapies is needed in the future. By weaving gut-centric therapeutics into the management framework of heart failure, there is potential to move beyond symptomatic control toward modifying upstream drivers of inflammation, remodeling, and progression. Such an approach may complement and offer a new frontier in the management of a disease that continues to challenge clinicians.

## Figures and Tables

**Table 1:** Key clinical studies of gut-derived biomarkers in heart gailure.

Biomarker	Study	N	HF Type	Key Finding	HR (95% CI)	Follow-Up
TMAO	Tang et al., 2014 [42]	720	Stable HF	TMAO independently predicts mortality.	2.2 (1.42–3.43) *p* < 0.001	5 years
	Suzuki et al., 2016 [11]	972	Acute HF	TMAO are strongly associated with poor prognosis and higher mortality risk.	1.16 (1.01–1.33) *p* = 0.037.	1 year
Endotoxin (LPS)	Sandek et al., 2007 [27]	22 CHF/22 controls	Chronic HF (NYHA II–IV)	Elevated serum IgA-anti-LPS antibodies in the HF cohort due to gut barrier disruption and index of chronic inflammation.	Cross-sectional	N/A
	Krack et al., 2005 [34]	Review	Mixed HF	LPS correlates with HF severity and inflammation.	Narrative review	N/A
Microbiome dysbiosis	Jie et al., 2017 [54]	218 ASCVD /187 controls	Atherosclerosis	Depletion of *Roseburia* and *F. prausnitzii*, and overgrowth of *Enterobacteriaceae* and *Strep* sp. in CVD. Enriched TMA and LPS in ASCVD cohort.	Metagenomic analysis Cross-sectional	N/A

## Data Availability

No new data were created or analyzed in this study.
